# Metabolomic Profiling of Mice Serum during Toxoplasmosis Progression Using Liquid Chromatography-Mass Spectrometry

**DOI:** 10.1038/srep19557

**Published:** 2016-01-20

**Authors:** Chun-Xue Zhou, Dong-Hui Zhou, Hany M. Elsheikha, Yu Zhao, Xun Suo, Xing-Quan Zhu

**Affiliations:** 1 State Key Laboratory of Veterinary Etiological Biology, Key Laboratory of Veterinary Parasitology of Gansu Province, Lanzhou Veterinary Research Institute, Chinese Academy of Agricultural Sciences, Lanzhou, Gansu Province 730046, PR China; 2 National Animal Protozoa Laboratory and College of Veterinary Medicine, China Agricultural University, Beijing 100193, PR China; 3 Faculty of Medicine and Health Sciences, School of Veterinary Medicine and Science, University of Nottingham, Sutton Bonington Campus, Loughborough, LE12 5RD, UK

## Abstract

Better understanding of the molecular changes associated with disease is essential for identifying new routes to improved therapeutics and diagnostic tests. The aim of this study was to investigate the dynamic changes in the metabolic profile of mouse sera during *T. gondii* infection. We carried out untargeted metabolomic analysis of sera collected from female BALB/c mice experimentally infected with the *T. gondii* Pru strain (Genotype II). Serum samples were collected at 7, 14 and 21 day post infection (DPI) from infected and control mice and were subjected to liquid chromatography-quadrupole time-of-flight mass spectrometry (LC-Q-TOF-MS)-based global metabolomics analysis. Multivariate statistical analysis identified 79 differentially expressed metabolites in ESI+ mode and 74 in ESI− mode in sera of *T. gondii*-infected mice compared to the control mice. Further principal component analysis (PCA) and partial least squares-discrimination analysis (PLS-DA) identified 19 dysregulated metabolites (5 in ESI+ mode and 14 in ESI− mode) related to the metabolism of amino acids and energy metabolism. The potential utility of these metabolites as diagnostic biomarkers was validated through receiver operating characteristic (ROC) curve analysis. These findings provide putative metabolite biomarkers for future study and allow for hypothesis generation about the pathophysiology of toxoplasmosis.

*Toxoplasma gondii* is an obligate intracellular protozoan pathogen that can infect any warm-blooded animal species and virtually any nucleated cell types, which makes *T. gondii* a highly prevalent parasitic infection with up to one third of the world’s human population has been estimated to be infected^1^. While infection in immunocompetent people is asymptomatic, *T. gondii* can cause retinochoroiditis and encephalitis in immunocompromised individuals, and has been a frequent cause of abortion or congenital toxoplasmosis in pregnant women^2^. Variation in the clinical manifestations and severity of the disease is determined by both the host’s genetic background and the genotype of the infecting parasite strain^3^. Type I strains are lethal (LD100~1), and in contrast, type II and III strains are significantly less virulent (LD100 ≥ 10^3^)^4^. In North America and Europe, Type II strains are responsible for human toxoplasmosis and chronic infections in food animals^5^.

The prognosis of toxoplasmosis largely depends on the stage of *T. gondii* infection. Acute infection is caused by the tachyzoite stage of the parasite, and as infection progresses tachyzoite transforms to the bradyzoite form, which is associated with long-term chronic infection, where the parasite persists for many years or the life-time of the host^6^. *T. gondii* strictly relies on host cellular metabolism to obtain the energy and biosynthetic building blocks required for their replication^7^. But, very little is known about the physiological state and biochemical landscape of both the host and parasite during these acute and chronic infections. During chronic infection *T. gondii* enters a quiescent state; however there is a possibility that even the latent parasite stage exerts a considerable impact on the host metabolic profiles^8^. The alteration in the metabolism of the host during acute and latent stages of *T. gondii* infection needs to be defined in order to better understand the pathogenesis of toxoplasmosis (e.g., the potential role of quiescence in combating host parasiticidal responses) and develop drugs that target processes essential for parasite survival during latency.

How an intracellular pathogen such as *T. gondii* manipulates the metabolism of its host in order to create a more hospitable metabolic niche has been a central question in toxoplasmosis research^9^. *T. gondii* is in a constant competition with its host over access to carbon sources and nutrients because this parasite needs to tailor its metabolism to fulfill particular tasks, such as the synthesis of virulence factors or the maintenance of its energy/redox state in hospitable host environments. Recently, a pioneering study has investigated the metabolic needs and capabilities of *T. gondii* using a combined computational and experimental approach^10^. The interplay between *T. gondii* parasites and their hosts has been studied for decades using targeted approaches, such as the analysis of mutants or host immunological responses^11^,^12^. Although much has been learned from such studies, they focus on individual pathways and fail to reveal the global effects of infection on the host’s metabolism. To address this limitation, high-throughput methods, such as transcriptomics and proteomics, have been used to study host- *T. gondii* interactions^13^,^14^. However, the effect of *T. gondii* on the biochemical composition of host body fluids remains unknown. *T. gondii,* although strictly intracellular it has been detected in body fluids^15^,^16^. These extracellular niches may provide different nutrient availability or other benefits and challenges. Therefore, a complete understanding of *T. gondii* pathogenesis necessitates consideration of the effect of the parasite on the metabolism of body fluids over time. In this regard, host serum offers an important window for understanding the biological changes that occur in host during infection.

Recently, techniques that detect and quantify multiple small chemical metabolites in complex biological samples have been developed, giving rise to the field of metabolomics^17^. Metabolomic studies have relied on the use of analytical platforms, such as nuclear magnetic resonance (NMR), gas or liquid chromatography coupled to mass spectrometry (GC-MS/LC-MS) and capillary electrophoresis/mass spectrometry (CE-MS) for the analysis of the metabolome^7^. Among these techniques, LC/MS has been widely used in metabolites’ identification and quantification, and has been a useful metabolomic method with high sensitivity, peak resolution, and reproducibility^18^. Also, it can identify and quantify hundreds of metabolites with higher mass accuracy, thus allowing comprehensive metabolic profiling^19^. This non-targeted metabolomic approach enables changes in many metabolites to be evaluated in an unbiased fashion, and thus can provide a global view of dynamic metabolic variations, simulating the changes that occur during the disease development, which can allow the discovery of key metabolites that are able to discriminate healthy individuals from patients^20^.

We used LC-Q/TOF-MS-metabolomic-based approach to investigate the impact of *T. gondii* infection on host metabolism using BALB/c mice as a model. To our knowledge, this is the first comprehensive metabolomic analysis of the effect of *T. gondii* infection on host serum metabolism. Our findings indicate that LC-MS-based untargeted metabolomic analysis is able to identify dysregulated metabolites in mouse serum and discriminate the metabolomic profile between *T. gondii*-infected and non-infected mice, as well as during the different stages of the disease. A few mammalian signaling pathways of fundamental importance for host metabolic and immune homeostasis have been affected, including pathways involved in host amino acid, lipid and energy signaling. The disruption of such pathways reveals the significant impact of *T. gondii* infection on host metabolic function and may shed light on the molecular mechanisms used by *T. gondii* to cause disease, as well as those used by host defenses.

## Results

### Characteristics of infected mice

**Figure 1:**
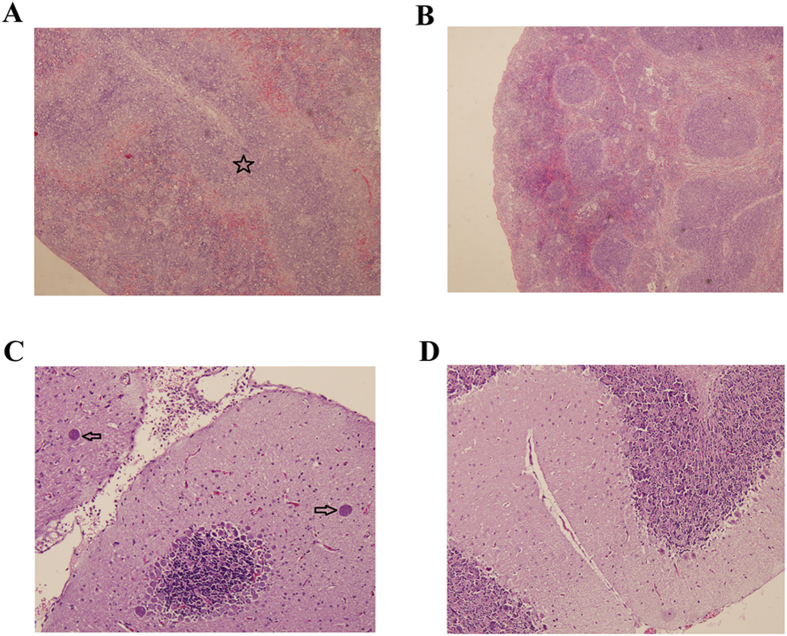
Histopathological lesions in mouse tissues infected with *Toxoplasma gondii* Pru strain (10 cysts per mouse by oral route, H&E stain). (**A**) A section of spleen from infected mice at 14 DPI. Pentagram indicates the largely scattered white pulp; (**B**) A section of spleen from normal mice at 14 DPI showing no histological abnormalities; (**C**) Brain histology of infected mice at 21 DPI. Arrow indicates the tissue cyst; (**D**) Brain histology of normal mice at 21 DPI without any pathological changes. Magnifications: 40X (**A,B**); 100X (**C,D**).

Control mice did not exhibit any clinical signs during the entire course of infection. However, infected mice showed time-dependent increase in the progression of illness. Infection has been assessed via the detection of *T. gondii* B1 gene in various mouse tissues and all infected mice were tested positive (data not shown). At 7 DPI mice showed mild signs, which progressed to typical clinical signs of acute infection at 10 DPI. By 14 DPI mice began to recover and at 21 DPI all mice restored their normal physical state. Using H&E staining, spleen and brain samples were examined for histopathological damages caused by *T. gondii*. As expected, all control mice tissues did not show any obvious pathological changes. In contrast, infected mice showed splenomegaly at 14 DPI (Fig. S1). Also, a reduced white pulp with an expansion of the red pulp compartment was observed at 14 DPI (Fig. 1A). Brain of infected mice had inflammatory reactions characterized by focal mononuclear cell infiltrates and glial nodules at 14 and 21 DPI. A number of multifocally distributed tissue cysts filled with bradyzoites were detected at 21 DPI (Fig. 1C).

### Comparative metabolite profiling of serum from infected mice and controls

**Figure 2:**
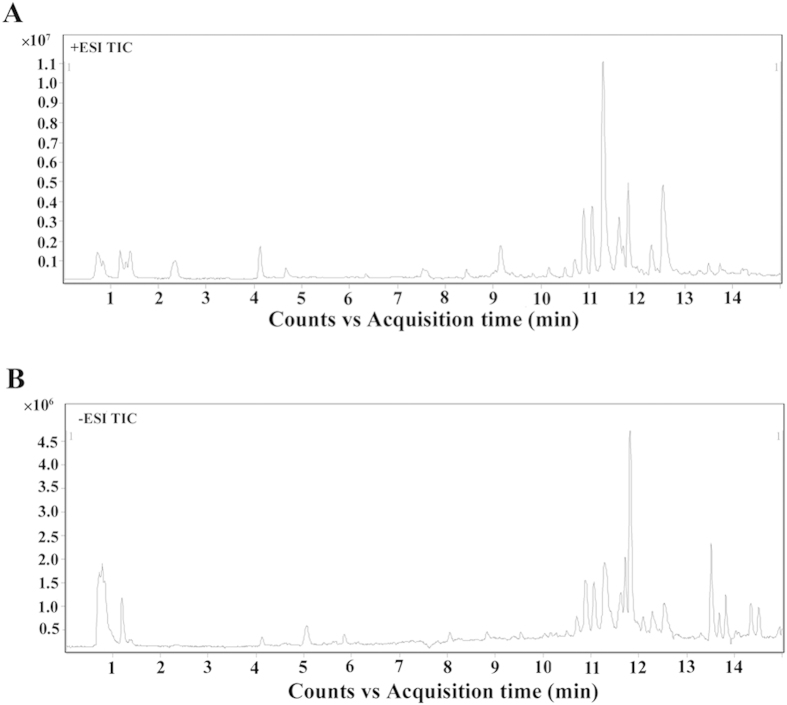
Representative total ion current (TIC) chromatograms of infected mice serum obtained in (**A**) positive ion mode (ESI+) and (**B**) negative ion mode (ESI-). Y-axis represents the intensity.

We next analyzed all total ion chromatograms of mice sera. We noted a stable retention time without peaks’ drifts (Fig. S2). Representative TIC chromatograms of serum samples from infected mice are shown (Fig. 2). Baseline correction, peak deconvolution, alignment, normalization and median-centering are applied to process the data, and there are 509 and 532 retention time-exact mass pairs determined in each sample profile analyzed in positive or negative ion mode, respectively. The majority of peaks in the chromatograms are identified as endogenous metabolites and most of these metabolites are involved in multiple biochemical processes, especially in energy and lipid metabolism. The LC–MS system stability and reproducibility for the large-scale sample analysis were observed in the PCA scores plot representation of QC samples by using the metabolite profiles obtained in positive and negative ion modes, as shown in Fig. S3. Six QC samples were run for serum samples throughout the entire analysis. There was no drift in the PCA scores plot representation of QC samples, and 87.2% and 93.2% of RSDs were less than 30% for serum samples in the positive mode and negative mode, respectively. Therefore, the LC–MS system repeatability and stability were deemed acceptable.

To investigate the metabolic variations during the disease progression, all observations acquired in both ion modes were first analyzed using two components PCA score trajectories plot (Fig. S4). The score plot did not illustrate good classification of the infected mice groups and their corresponding control groups. Subsequently, the cross-validated two-component PLS-DA models were used for further multivariate analysis, and showed a satisfactory modeling. To reduce the system error and interference of impurity peak, the OPLS-DA model of SIMCA-P were then established for the following analysis.

### Temporal variation in metabolic profiling

**Figure 3:**
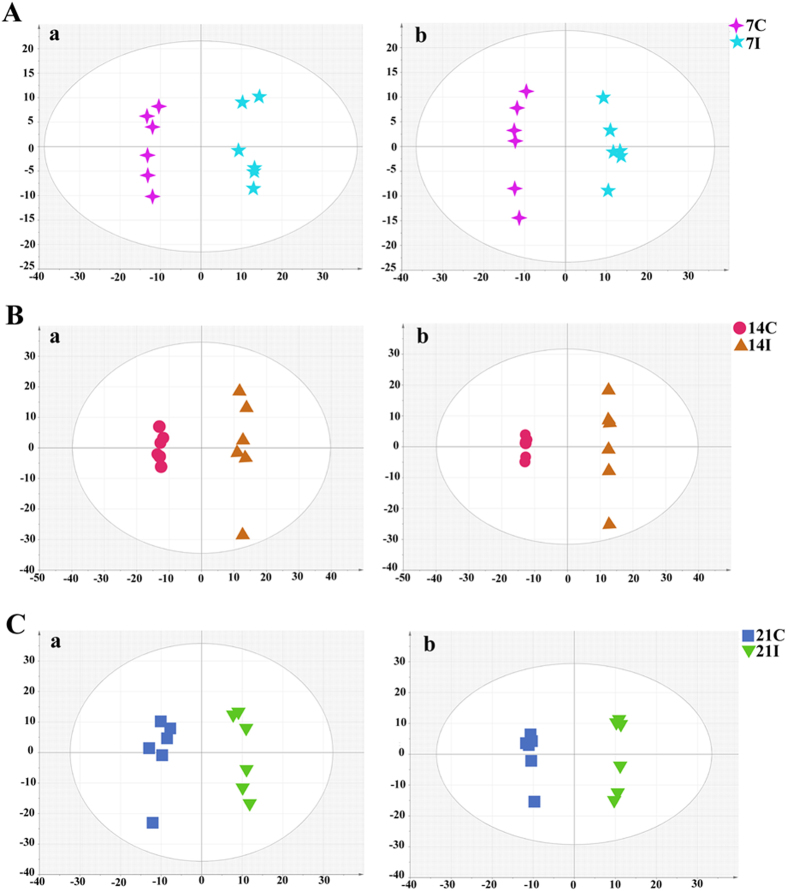
OPLS-DA score plot of *T. gondii* infected and control groups. (**A**) The 7D infected group (7I) and the 7D control group (7C) (R^2^X = 0.427, R^2^Y = 0.985,Q^2^ = 0.829 ESI+; R^2^X = 0.392, R^2^Y = 0.987, Q^2^ = 0.801 ESI−); (**B**) The 14D infected group (14I) and the 14D control group (14C) (R^2^X = 0.699, R^2^Y = 0.996, Q^2^ = 0.934 ESI+; R^2^X = 0.771, R^2^Y = 1, Q^2^ = 0.944 ESI−); (**C**) The 21D infected group (21I) and the 21D control group (21C) (R^2^X = 0.506, R^2^Y = 0.975,Q^2^ = 0.877 ESI+; R^2^X = 0.505, R^2^Y = 0.997, Q^2^ = 0.852 ESI−). In the OPLS-DA score plot, each data point represents one mouse serum sample, and the distance between points in the plot indicates the similarity between samples. (**a**) ESI+; (**b**) ESI-. x- and y-axes represent PC1 and PC2, respectively.

**Figure 4:**
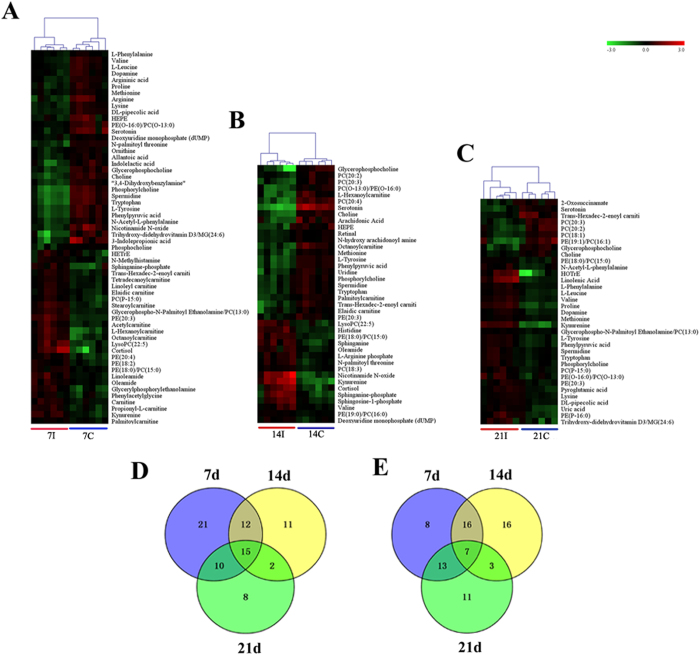
Comparison of the mice serum metabolomes during the course of infection. Heat maps representing the significantly changed metabolites between infected groups and the corresponding control groups in ESI+ mode (**A–C**). Individual samples are separated using hierarchical clustering, with the dendrogram scaled to represent the distance between each branch. Heat maps show a clear separation of metabolomic profile between different groups. Normalized signal intensities (log2 transformed and row adjustment) are visualized as a color spectrum and the scale from least abundant to highest ranges is from −3.0 to 3.0. Green indicates low expression, whereas red indicates high expression of the detected metabolites. (**A**) 7D infected group vs 7D control; (**B**) 14D infected group vs 14D control; (**C**) 21D infected group vs 21D control; (**D**) Venn diagram shows a clear separation among sample groups at the three indicated infection time points in the ESI + mode; (**E**) Venn diagram shows a clear separation among sample groups at three indicated infection time points in the ESI- mode.

To better define the metabolic variations in mice serum after *T. gondii* infection, all observations acquired in both ion modes were analyzed using OPLS-DA. Generally, the model is believed to be reliable when the Q^2^ > 0.4. As shown in Fig. 3 the scores plots of OPLS-DA model discriminated the infected groups from their corresponding control groups, which exhibited satisfactory classification. The differential metabolites at different times post infection were selected according to the VIP threshold (VIP > 1) in the OPLS-DA model and the *p*-value of student’s *t*-test (*p* < 0.05) after FDR correction. As shown in Fig. 4A–C the serum metabolic states of the infected mice deviated significantly from their corresponding controls in the ESI+ mode. The serum metabolic profiling in the infected mice changed most significantly from their controls at 7 DPI. As the infection time increases the number of differential metabolites decreased, indicating a restoration of the dysregulated metabolic state in a time-dependent manner. Meanwhile, metabolic profile observed in ESI- mode also exhibited the same trend (Fig. S5). Of the 79 differential metabolites detected in ESI+ mode, 43 were shared between at least two of the three infection stages (Fig. 4D). Also, we detected 74 significant differential metabolites in ESI- mode, of which 43 were shared between at least two of three infection stages (Fig. 4E). As shown in Table 1, we detected 15 metabolites under ESI+ and 7 metabolites under ESI- that varied at all three time points. The related pathways of each metabolite were also listed by searching the KEGG pathway database (http://www.genome.jp/kegg/), and the majority of these 22 metabolites were involved in lipid metabolism and amino acid metabolism.

### Metabolic Variations between different stages

**Figure 5:**
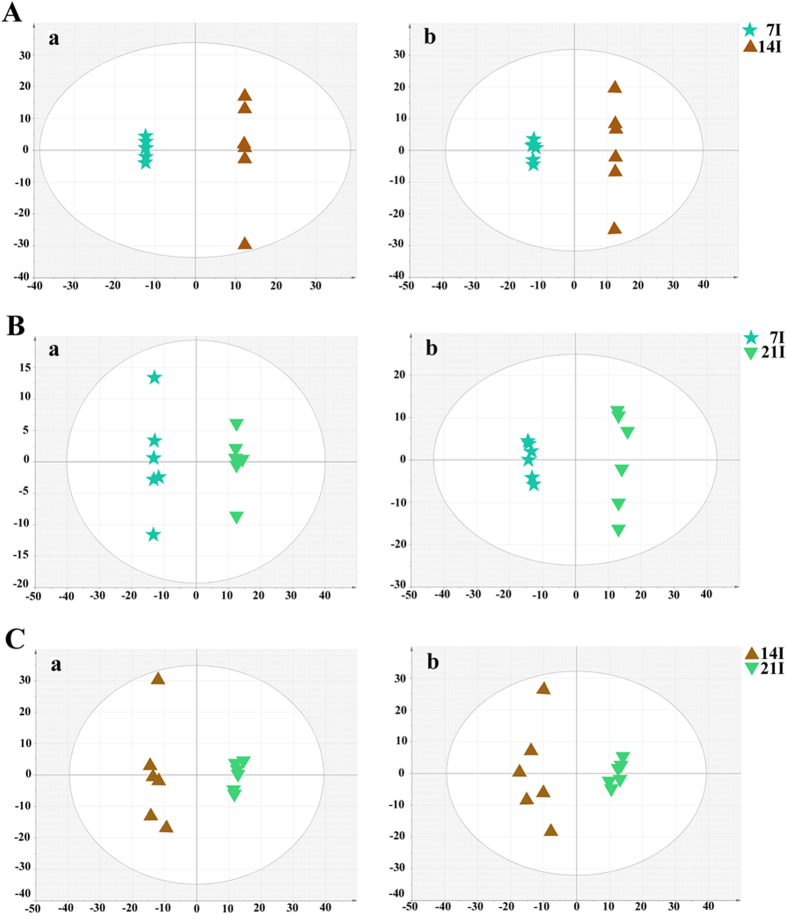
OPLS-DA score plot showing a clear separation between the infected serum samples at different time points after infection. (**A**) The 7D infected group vs the 14D infected group (R^2^X = 0.782, R^2^Y = 1, Q^2^ = 0.953 ESI+; R^2^X = 0.717, R^2^Y = 1, Q^2^ = 0.946 ESI−); (**B**) The 21D infected group vs the 7D infected group (R^2^X = 0.433, R^2^Y = 0.997, Q^2^ = 0.952 ESI+; R^2^X = 0.517, R^2^Y = 0.996, Q^2^ = 0.954 ESI−); (**C**) The 21D infected group vs the 14D infected group (R^2^X = 0.604, R^2^Y = 0.988, Q^2^ = 0.93 ESI+; R^2^X = 0.539, R^2^Y = 0.957, Q^2^ = 0.808 ESI−). (**a**) ESI+, (**b**) ESI−. X axis is PC1, Y axis is PC2.

**Figure 6:**
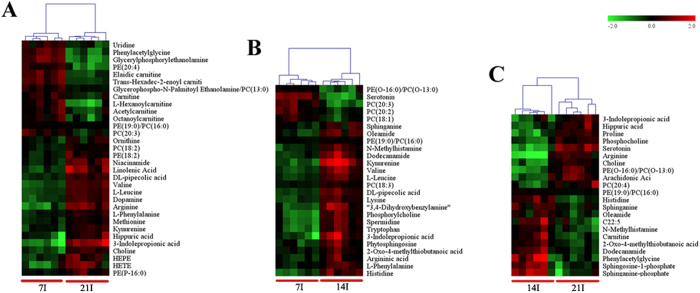
Heat map showing the significantly dysregulated metabolites identified between different infected groups in ESI+ mode. Individual samples are separated using hierarchical clustering, with the dendrogram scaled to represent the distance between each branch. Heat maps show a clear separation of the metabolomic profile between different infected groups. Normalized signal intensities (log2 transformed and row adjustment) are visualized as a color spectrum and the scale from least abundant to highest ranges is from −2.0 to 2.0. Green color indicates low expression, and red color indicates high expression of the detected metabolites. (**A**) 7D infected group vs 21D infected group; (**B**) 7D infected group vs 14D infected group; (**C**) 14D infected group vs 21D infected group.

To further our understanding of the effects of *T. gondii* infection on host serum metabolism, we examined the changes in the chemical composition of serum extracts during different stages of the infection as defined by histopathology. As shown in Fig. 5 score plot from supervised OPLS-DA showed good discrimination between different infected animal groups in both positive and negative ion modes, which indicated that their metabolic profiles were distinct. Those significantly changed metabolites were then used to construct heat maps for unsupervised clustering. As shown in Fig. 6, heat maps of the differential metabolites detected in ESI+ mode showed a clear clustering for each group in agreement with the OPLS-DA results. Mice at 14 and 21 DPI were recovering from the acute infection, and exhibited the minimal difference in the number of differential metabolites (Fig. 6C). At 7 DPI mice would be soon developing an acute infection stage, exhibited the larger number of differential metabolites when compared with other two infected groups (Fig. 6A,B). We also constructed heat maps using differential metabolites detected in ESI- mode. Even though sample clusters overlapped slightly, most samples were clearly grouped into two differentiated clusters and subsequent statistic analysis revealed dramatic time-dependent changes in the number of differential metabolites (Fig. S6).

### Identification of potential biomarkers

**Figure 7:**
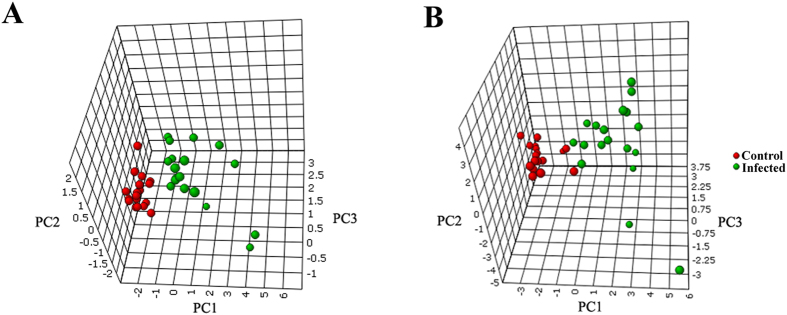
3D plot showing clear separation between *T. gondii* infected samples and controls using PCA analysis in ESI+ mode (**A**) and ESI- mode (**B**).

The potential biomarker metabolites that significantly contributed to the discrimination of infected mice from controls were identified by using the receiver operating characteristic (ROC) curve analysis in MetaboAnalyst 3.0^21^. Firstly, univariate ROC curve analyses were applied to quantify the predictive performance of each potential biomarker. The sensitivity and specificity trade-offs were calculated for each selected metabolite using the area under the ROC curve (AUC). This analysis identified 5 significantly differential metabolites in ESI+ mode and 14 significantly differential metabolites in ESI- mode with AUC > 0.8, which were selected after standardization of data as shown in Table 2. 3D PCA models and heat maps were constructed using the marker metabolite intensities as variables. As shown in Fig. 7 and Fig. S7, the infected mice group and the control group were separated into two different regions.

**Figure 8:**
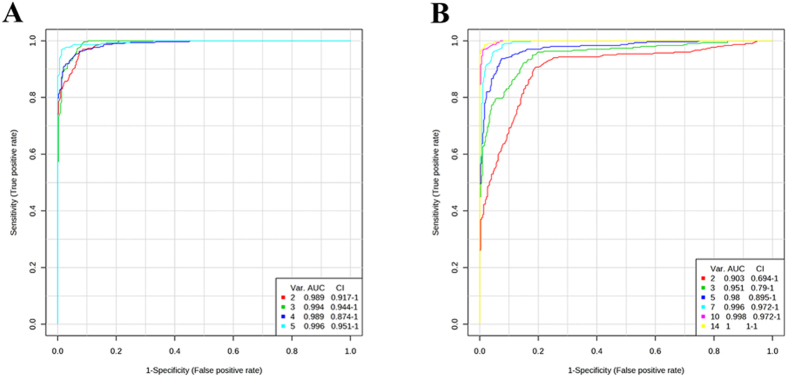
Comparisons of different matabolites panels based on ROC curves. ROC curves are generated by Monte-Carlo cross validation (MCCV) using balanced subsampling. PLS-DA algorithm was selected as classification and feature ranking method. In each MCCV, two-thirds of the samples are used to evaluate the feature (metabolite) importance. Different panels of important features are then used to build classification models, which is validated on the one-third samples that were left out. The different models with specific feature numbers and their corresponding AUCs are shown on the figure. (**A**) Marker metabolites detected in ESI+ mode, (**B**) Maker metabolites detected in ESI- mode. Var. (variables) indicates the number of selected features.

To evaluate the utility of metabolite combination for the diagnosis of *T.gondii* infection, multivariate exploratory ROC analysis was performed. ROC curves are generated by Monte-Carlo cross validation (MCCV) using balanced subsampling. Different biomarker modules were created through the built-in feature selections. As shown in Fig. 8, the AUC values obtained in this analysis were 0.996 (95% C.I. 0.951-1) and 1(95% C.I. 1-1) for all the 5 marker metabolites in ESI+ mode and 14 marker metabolites in ESI- mode, respectively (an ideal model would have an AUC of 1). Together, these results indicate that the indentified potential biomarkers, alone or in combination, can discriminate the infected samples from the controls with high accuracy and could be powerful indicators for Toxoplasmosis monitoring.

## Discussion

Host-pathogen interactions have been studied using several “omics” techniques, such as genomics, transcriptomics, and proteomics, and more recently metabolomics, a newly established omics’ methodology based on the analysis of small metabolites to study chemical changes in biological properties^22^. The use of these omics methodologies has revealed important information about many biological systems and has greatly contributed to the development of systems biology. In the present study we performed a LC-Q/TOF-MS-based untargeted metabolomic study to investigate the global effect of *T. gondii* infection on the serum metabolism of BALB/c mice. Our results show that *T. gondii* infection causes significant dynamic changes in the metabolite balance of the serum of *T. gondii*-infected mice and identified novel biomarkers with potential diagnostic value.

Fifteen metabolites detected in ESI+ mode and 7 metabolites in ESI- mode were found to vary during the 21 day of the infection period (Table 1). They represent perturbations in multiple pathways, such as pyrimidine metabolism, lipid metabolism and tryptophan metabolism^23^. Among these metabolites, kynurenine, phosphatidylethanolamine (PE) (18:0)/phosphatidylcholine (PC) (15:0) were increased, whereas, the level of choline, glycerophosphocholine, serotonin, cytidine, N-Acetyl-DL-tryptophan, arachidonic acid and eicosatrienoic acid were decreased. These infection-specific metabolic alterations are consistent with the current understanding of host-*T. gondii* interaction biology. Metabolic function is known to influence the activity of the immune system^24^. In this regard, *T. gondii* infection induces major immunological changes, that in some cases are linked to the production of amino acid metabolites, such as kynurenine (consistent with our finding), a product of indoleamine 2,3 dioxygenase (IDO) enzymatic degradation of tryptophan; a key antiparasitic mechanism to control parasite growth^25^. The induction of IDO by interferon-γ (IFN-γ) and some pro-inflammatory cytokines is associated with depleted plasma tryptophan (in agreement with our finding), which may interfere with brain 5-HT synthesis, and increased production of anxiogenic and depressogenic tryptophan catabolites^26^. Evidence showed that kynurenine might be involved in depression and cognitive deficits^20^,^27^,^28^, which explains the depressive and anxiety symptoms exhibited by the infected mice. Serotonin is another metabolite in tryptophan metabolism and its depletion is also associated with depression, and anxiety^29^. The exact mechanism of this behavioral manipulation is unknown, but parasites cysts to disturb dopamine metabolism has been suggested as one of the most appealing causes^30^. A previous study showed that infection of mice by *T. gondii* caused a 14% increase in whole-brain dopamine levels upon establishment of chronic infection^31^. In agreement with this finding our study showed a increased level of serum DA in the infected mice compared to the controls only at 21 DPI (Fig. 4C), around the beginning of the chronic infection^32^. This time-dependent differential expression of DA may explain the link between abnormal behavioral alterations and *T. gondii* infection.

A clear metabolites’ separation has been observed not only between infected mice and the corresponding controls, but also between different infected mouse groups throughout the course of the infection (Fig. 6). The alterations in the serum metabolic profile of *T. gondii*-infected mice paralleled the clinical course of the disease, where the significant changes in metabolites were detected in infected mice at 7 DPI, followed by restoration of the metabolic profile to a normal level as infection proceeds to the chronic stage, indicating a re-balance of the disturbed metabolic state coinciding with regaining apparently normal physical status.

Previous applications of LC-MS based unbiased metabolomic technologies has enabled researchers to identify biomarkers for various diseases, such as cancer^33^,^34^ and other types of disease^35^,^36^, and to investigate host-parasite interaction and metabolic alterations in trypanosomiasis, schistosomiasis and malaria in a high-throughput manner^37^,^38^,^39^. In agreement with previous studies, our results demonstrated the power of the untargeted LC-MS-based metabolomic’s approach in profiling the metabolic changes in serum from *T. gondii*-infected mice. We identified 19 infection-specific metabolites, 5 in ESI+ mode and 14 in ESI- mode, in the serum samples. PCA models and heat maps constructed based on those selected metabolites showed reasonable separations between infected and control groups. ROC analysis supported the robustness of the PCA models by cross validation, in which all ROC value were high. Among these compounds, the 5 detected in ESI+ mode, including kynurenine, serotonin, glycerophosphocholine and choline and N-Acetyl-DL-tryptophan, showed higher sensitivity and specificity (Fig. 8). These compounds play critical roles in mediating host-pathogen interaction as described above. Most of the metabolites identified in ESI- mode decreased in the infected group, including oleic acid, eicosapentaenoic acid, arachidonic acid (the main precursor of eicosanoid hormones), HOME, N-Acetyl-DL-tryptophan, fumaric acid, citric acid, glutathione, malic acid and cytidine, probably due to the increased metabolomic demands during infection. Overall, these findings provide insight into the pathophysiology of *T. gondii* infection and have the potential to improve toxoplasmosis diagnosis and management.

## Methods

### Ethics statement

All animal procedures were approved by Animal Ethics Committee of Lanzhou Veterinary Research Institute, Chinese Academy of Agricultural Sciences (Permit No. LVRIAEC2014-002). All experiments were performed in strict compliance with the requirements of the Animal Ethics Procedures and Guidelines of the People’s Republic of China.

### Mice

Female BALB/c mice were purchased from Lanzhou University, China. All mice were bred and maintained under specific-pathogen-free (SPF) conditions in the experimental facility at Lanzhou University, where they received sterilized food and water *ad libitum*. All mice were kept in a temperature-controlled room (22 ± 0.5 °C) on reverse 12/12 h light/dark cycle. Mice were acclimated for about seven days prior to the start of the experiment and were weighed (weight >20 g) to establish a baseline for detecting any reduction in body weight caused by infection.

### Parasite strain, its maintainance, and mouse infection

*Toxoplasma gondii* type II Prugniuad (Pru) strain was maintained in mice via oral inoculation of cysts in mice. Cysts used in this study were isolated from brain tissues of infected BALB/c mice 40 days post-infection (DPI). Following anesthetizing animals, the brains of infected mice were removed and homogenized under sterile conditions. Then, the number of cysts was counted and diluted to 100 cysts/ml in phosphate buffered saline solution (PBS, pH7.2). Mice (6–8 weeks old) were infected with freshly prepared 10 cysts of *T. gondii* Pru strain by oral gavage. Mock-infected (control) mice underwent the identical infection protocol but with plain PBS without any parasite cysts. All mice were monitored for mortality and morbidity throughout the course of infection (i.e. 21 DPI).

### Confirmation of infection

Following anesthetizing animals, 6 mice from infected or control groups were sacrificed at 7, 14 and 21 DPI. Mice tissues, such as brain, blood, liver, spleen, lung, small intestine and kidney were collected and examined for the presence of *T. gondii*. Briefly, genomic DNA was extracted from these tissues using TIANamp Genomic DNA kit (TianGen™, Beijing, China) according to the manufacturer’s instructions. Then, a PCR targeting the *T. gondii* B1 gene was performed to detect infection using the specific primers (5′-AAC GGG CGA GTA GCA CCT GAG GAG-3′ and 5′-TGG GTC TAC GTC GAT GGC ATG ACA AC-3′)^40^. Positive control (DNA from parasites) and negative control (PBS) samples were included in each test. Infection was also confirmed by histopathological examination of brain and spleen collected from the mice after euthanasia. Tissue samples were fixed in 10% neutral buffered formalin solution for 2 weeks and then were dehydrated by gradually soaking in alcohol and xylene and embedded in paraffin. The paraffin-embedded specimens were cut into 5-μm sections, stained with hematoxylin-eosin (H&E), and examined under a digital optical microscope (Olympus, Tokyo, Japan).

### Non-targeted metabolic profiling and spectral acquisition

The blood samples from different infection groups (7d, n = 6; 14d, n = 6; 21d, n = 6) and their corresponding control groups (n = 6) were prepared as described previously^41^. Blood was collected by retro-orbital bleed into Eppendorf tubes directly and allowed to clot for 30 min followed by centrifugation at 3000 *g* for 10 minutes at 4 °C to collect the serum fraction. Supernatants were frozen immediately at −80 °C and shipped on dry ice. A total of 100 μl serum sample was thawed on ice at 4 °C and then precipitated by a solvent of acetonitrile-water (7:3), treated with ultrasonic wave for 15 min. The mixture was centrifuged at 12,000 rpm for 15 min at 4 °C and then the supernatant was transferred to 1.5 ml polypropylene tube.

Metabolomic analysis was performed using an Agilent 1290 Infinity LC System coupled to an Agilent 6530 Q-TOF/MS (Agilent Corp.) (LC-Q/TOF-MS) equipped with an electrospray ionization (ESI) source. The separation of all samples was performed on a C18 column (Agilent Technologies, Santa, Clara, CA) (dimension 100 × 2.1 mm, 1.8 μm particle size, 100 Å pore size) with the column temperature maintained at 40 °C. The LC-MS system was run in a binary gradient solvent mode consisting of 0.1% (v/v) formic acid in water (solvent A) and 0.1% formic acid in acetonitrile (solvent B) and the flow rate was 0.4 ml/min. The linear gradient is described in Table 3.

The sample injection volume was 4 μl. Sample analysis was carried out in both positive and negative ion modes. The mass scanning range was 50−1000 m/z. Nitrogen was used as the dry gas and cone gas with parameters described in Table 4. The mass spectrometry was operated in V optics mode and the data acquisition rate was set to 0.03 s, with interscan delay of 0.02 s. To ensure accuracy and reproducibility, LockSpray was used in all analyses and Leucine-enkephalin was selected as the lockmass for positive ([MH]^+^ = 556.2771) and negative ([MH]^−^ = 554.2615) ion modes.

We used Quality control (QC) samples to assess the reproducibility and reliability of the LC-MS system. QC samples were prepared by mixing equal volumes (10 μl) from each serum sample as they were aliquoted for analysis. This “pooled” sample was used to provide a “mean” profile representing all the analytes encountered during the analysis^42^. The pooled “QC” sample was injected five times at the beginning of the run to ensure system equilibrium and then every 6 samples to further monitor the stability of the analysis^43^.

### Data processing and statistics

Raw data were acquired by using LC-MS-Q-TOF MassHunter Workstation QTOF Acquisition software (B.03.01) (Agilent Technologies, Santa Clara, CA, USA) in an untargeted mode. LC-MS raw data file was converted into common format, and then directly processed by the XCMS toolbox (http://metlin.scripps.edu/xcms/)^44^. Peak picking, peak grouping and retention time correction were all involved in data pre-treatment, which was done through the XCMS software that was implemented with the freely available R programming language (v 2.13.1). Retention time (RT)-m/z data pairs were used to identify ion intensities of those detected peaks. The consistent variables (ion intensity information) were obtained by filtering peaks with 80% missing values and those with isotope ions from each group.

In order to remove the offsets and adjust the importance of high and low abundance metabolites to an equal level, the data was pre-processed by both mean-centering and variance-scaling prior to multivariate analysis. Then, the resulting three-dimensional matrix, which includes assigned peak indices (retention time-m/z pairs), sample names and variables, was further subjected to multivariate data analysis.

The resulting scaled datasets were imported into SIMCA-P + 11.0 (Umetrics, Umea, Sweden) where multivariate analyses such as principal components analysis (PCA) and orthogonal partial least-squares-discriminant analysis (OPLS-DA) were carried out to investigate and visualize the pattern of metabolite changes in an un-supervised and supervised manner, respectively. PCA was used to validate quality of the analytical system performance and to observe possible outliers. OPLS-DA was applied to obtain an overview of the complete data set and discriminate the variables that are responsible for variation between the groups. The quality of the models was evaluated with the relevant R^2^ and Q^2^ as well discussed elsewhere^45^. The differential metabolites were selected when the statistically significant threshold of variable influence on projection (VIP) values obtained from the OPLS-DA model was larger than 1.0. Meanwhile, the *p* values from a two-tailed Student’s *t-*test on the normalized peak areas were less than 0.05^46^. Log 2 fold change (FC) was used to show how these selected differential metabolites varied between groups. The dataset of selected differential metabolites was imported into MetaboloAnalyst 3.0 for heatmap generation and multivariate statistics. The areas (AUC) under the receiver operating characteristic curves (ROC) were constructed to evaluate the effectiveness of potential biomarkers. Results were considered significant when *p* value is less than 0.05.

Putative metabolites were first derived by searching the exact molecular mass data from redundant m/z peaks against the online HMDB (http://www.hmdb.ca/), METLIN (http://metlin.scripps.edu/) and KEGG (www.genome.jp/kegg/) databases. A specific metabolite was sieved out when a match with a difference between observed and theoretical mass was less than 10 ppm. Then, the metabolite molecular formula of matched metabolites was further identified by the isotopic distribution measurement^47^.

## Additional Information

**How to cite this article**: Zhou, C.-X. *et al.* Metabolomic Profiling of Mice Serum during Toxoplasmosis Progression Using Liquid Chromatography-Mass Spectrometry. *Sci. Rep.* **6**, 19557; doi: 10.1038/srep19557 (2016).

## Supplementary Material

Supplementary Information

## Figures and Tables

**Table 1:** List of dysregulated metabolites identified in the serum of mice throughout the entire infection process.

Modes	mz	Name	*R*t (min)	7d*	14d*	21d*	Metabolic pathways
ESI+	104.1072	Choline	0.78	−1.16	−1.20	−0.35	Glycine, serine and threonine metabolism; Glycerophospholipid metabolism; bile secretion
	258.1101	Glycerophosphocholine	0.81	−0.95	−1.54	−0.78	Glycerophospholipid metabolism
	118.0863	Valine	1.2	−0.65	0.53	0.63	Valine, leucine and isoleucine degradation and biosynthesis
	150.0584	Methionine	1.21	−0.35	−0.71	0.25	Cysteine and methionine metabolism
	182.0812	L-Tyrosine	1.25	−1.18	−1.08	0.58	Tyrosine metabolism; Phenylalanine metabolism; Phenylalanine, tyrosine and tryptophan biosynthesis; Thiamine metabolism
	165.0544	Phenylpyruvic acid	1.25	−1.19	−1.04	0.58	Phenylalanine metabolism; Phenylalanine, tyrosine and tryptophan biosynthesis
	177.1022	Serotonin	1.79	−1.06	−3.30	−0.73	Tryptophan metabolism; bile secretion
	209.092	Kynurenine	2.27	0.83	2.72	1.80	Tryptophan metabolism
	170.0598	Phosphorylcholine	4.12	−1.69	−0.62	0.38	Glycerophospholipid metabolism
	146.0601	Spermidine	4.13	−1.67	−0.57	0.34	Not known
	205.0972	Tryptophan	4.13	−1.60	−0.66	0.37	Phenylalanine, tyrosine and tryptophan biosynthesis
	398.3268	Trans-Hexadec-2-enoyl carnitine	10.01	0.46	−1.03	−0.93	Not known
	482.3244	PE (18:0)/PC (15:0)	10.72	0.36	1.05	0.27	Not known
	440.3137	PE (O-16:0)/PC (O-13:0)	11.51	−0.66	−0.98	0.65	Not known
	504.3066	PE(20:3)	12.44	1.14	−0.43	0.31	Not known
ESI−	242.0798	Cytidine	0.81	−0.66	−0.73	−0.71	Pyrimidine metabolism
	180.0666	L-Tyrosine	1.27	−1.23	−0.49	0.77	Tyrosine metabolism
	130.0873	L-Leucine	1.42	−0.51	1.14	0.79	Valine, leucine and isoleucine degradation; Valine, leucine and isoleucine biosynthesis
	245.0933	N-Acetyl-DL-tryptophan	6.14	−2.25	−2.11	−1.18	Not known
	508.3408	1-heptadecanoyl-sn-glycero-3-phosphocholine	12.56	−0.21	0.48	0.14	Not known
	303.2328	Arachidonic Acid	13.69	−0.65	−1.17	−0.72	Arachidonic acid metabolism
	305.2488	Eicosatrienoic acid	14.1	−0.60	−1.14	−0.49	Not known

^*^indicates Log2 Fold change.

**Table 2:** Potential marker metabolites detected in LC/MS chromatograms.

Ion mode	Metabolites	*R*t (min)	*P*-value	AUC	Log2 (FC)	Metabolic pathways
ESI (−)	Oleic Acid	14.51	1.14E-03	0.827	−0.67	Fatty acid biosynthesis
ESI (−)	Eicosapentaenoic Acid	14.29	1.31E-05	0.88	−0.88	Biosynthesis of unsaturated fatty acids
ESI (−)	Arachidonic Acid	13.69	9.90E-06	0.889	−0.83	Arachidonic acid metabolism
ESI (−)	HOME	13.58	5.84E-06	0.886	−0.85	Not known
ESI (−)	PC (15:0)	12.45	6.75E-05	0.898	0.52	Not known
ESI (+)	Glycerophospho-N-Palmitoyl Ethanolamine	11.22	2.70E-03	0.836	0.27	Not known
ESI (−)	PE (16:0)	11.22	2.10E-04	0.867	0.32	Not known
ESI (−)	Taurocholic acid	8.05	1.01E-03	0.802	1.26	Taurine and hypotaurine metabolism; bile secretion
ESI (−)	N-Acetyl-DL-tryptophan	5.95	2.71E-07	0.963	−1.94	Not known
ESI (−)	L-Phenylalanine	2.34	1.29E-04	0.843	0.33	Aromatic amino acid metabolism
ESI (+)	Kynurenine	2.27	1.54E-04	0.997	1.77	Tryptophan metabolism
ESI (+)	Serotonin	1.79	3.52E-10	0.975	−1.69	Tryptophan metabolism
ESI (−)	Fumaric acid	1.36	1.12E-03	0.923	−0.77	TCA cycle
ESI (−)	Citric acid	1.21	5.01E-05	0.855	−0.62	TCA cycle
ESI (−)	Glutathione, oxidized	1.21	1.22E-03	0.824	−0.87	Glutathione metabolism; Methane metabolism
ESI (−)	Malic acid	1.17	1.10E-02	0.883	−0.69	Not known
ESI (+)	Glycerophosphocholine	0.81	8.48E-09	0.978	−1.37	Glycerophospholipid metabolism
ESI (−)	Cytidine	0.81	4.01E-05	0.882	−0.73	Pyrimidine metabolism
ESI (+)	Choline	0.78	6.00E-07	0.938	−0.93	Glycerophospholipid metabolism

**Table 3:** The gradient of mobile phase.

Time(min)	Flow rate (mL/min)	A (%)	B (%)
0	0.4	95	5
2	0.4	95	5
17	0.4	5	95
19	0.4	5	95

**Table 4:** Mass spectrometry parameters.

	Positive ion mode	Negative ion mode
Capillary voltage	4 kV	3.4 kV
Sampling cone	35 kV	50 kV
Source temperature	100 °C	100 °C
Desolvation temprature	350 °C	300 °C
Cone gas flow	50 L/h	50 L/h
Desolvation gas flow	600 L/h	700 L/h
Extraction cone	4 V	4 V

## References

[b1] Weiss L. M. & Dubey J. P. Toxoplasmosis: A history of clinical observations. Int. J. Parasitol. 39, 895–901 (2009). 19217908 10.1016/j.ijpara.2009.02.004 PMC2704023

[b2] Jones J. L., Kruszon-Moran D., Sanders-Lewis K. & Wilson M. *Toxoplasma gondii* infection in the United States, 1999–2004, decline from the prior decade. Am. J. Trop. Med. Hyg. 77, 405–410 (2007). 17827351

[b3] Grigg M. E., Ganatra J., Boothroyd J. C. & Margolis T. P. Unusual abundance of atypical strains associated with human ocular toxoplasmosis. J. Infect. Dis. 184, 633–639 (2001). 11474426 10.1086/322800

[b4] Dubey J. History of the discovery of the life cycle of *Toxoplasma gondii*. Int. J. Parasitol. 39, 877–882 (2009). 19630138 10.1016/j.ijpara.2009.01.005

[b5] Howe D. K. & Sibley L. D. *Toxoplasma gondii* comprises three clonal lineages: correlation of parasite genotype with human disease. J. Infect. Dis. 172, 1561–1566 (1995). 7594717 10.1093/infdis/172.6.1561

[b6] Yarovinsky F. Innate immunity to *Toxoplasma gondii* infection. Nat. Rev. Immunol. 14, 109–121 (2014). 24457485 10.1038/nri3598

[b7] Kafsack B. F. & Llinás M. Eating at the table of another: metabolomics of host-parasite interactions. Cell Host Microbe 7, 90–99 (2010). 20159614 10.1016/j.chom.2010.01.008 PMC2825149

[b8] Zhou C. X. *et al.* Global metabolomic profiling of mice brains following experimental infection with the cyst-Forming *Toxoplasma gondii*. PLoS One 10, e0139635 (2015). 26431205 10.1371/journal.pone.0139635 PMC4592003

[b9] Laliberte J. & Carruthers V. B. Host cell manipulation by the human pathogen *Toxoplasma gondii*. Cell. Mol. Life Sci. 65, 1900–1915 (2008). 18327664 10.1007/s00018-008-7556-x PMC2662853

[b10] Tymoshenko S. *et al.* Metabolic needs and capabilities of *Toxoplasma gondii* through combined computational and experimental analysis. PLoS Comput. Biol. 11, e1004261 (2015). 26001086 10.1371/journal.pcbi.1004261 PMC4441489

[b11] Leroux L.-P. *et al.* Parasite manipulation of the invariant chain (Ii/CD74) and the peptide editor H2-DM affects MHC-II antigen presentation during *Toxoplasma gondii* infection. Infect. Immun. 83, 3865–3880 (2015). 26195549 10.1128/IAI.00415-15 PMC4567656

[b12] Lagal V. *et al.* AMA1-deficient *Toxoplasma gondii* parasites transiently colonize mice and trigger an innate immune response that leads to long-lasting protective immunity. Infect. Immun. 83, 2475–2486 (2015). 25847964 10.1128/IAI.02606-14 PMC4432741

[b13] Nelson M. *et al.* Modulation of the host cell proteome by the intracellular apicomplexan parasite *Toxoplasma gondii*. Infect. Immun. 76, 828–844 (2008). 17967855 10.1128/IAI.01115-07 PMC2223483

[b14] Pittman K. J., Aliota M. T. & Knoll L. J. Dual transcriptional profiling of mice and *Toxoplasma gondii* during acute and chronic infection. BMC Genomics 15, 806 (2014). 25240600 10.1186/1471-2164-15-806 PMC4177681

[b15] Kupferschmidt O. *et al.* Quantitative detection of *Toxoplasma gondii* DNA in human body fluids by TaqMan polymerase chain reaction. Clin. Microbiol. Infect. 7, 120–124 (2001). 11318809 10.1046/j.1469-0691.2001.00224.x

[b16] Arantes T. P. *et al.* *Toxoplasma gondii*: Evidence for the transmission by semen in dogs. Exp. Parasitol. 123, 190–194 (2009). 19622353 10.1016/j.exppara.2009.07.003

[b17] Olszewski K. L. *et al.* Host-parasite interactions revealed by *Plasmodium falciparum* metabolomics. Cell Host Microbe 5, 191–199 (2009). 19218089 10.1016/j.chom.2009.01.004 PMC2737466

[b18] Zhou B., Xiao J. F., Tuli L. & Ressom H. W. LC-MS-based metabolomics. Mol. Biosyst. 8, 470–481 (2012). 22041788 10.1039/c1mb05350g PMC3699692

[b19] Theodoridis G., Gika H. G. & Wilson I. D. LC-MS-based methodology for global metabolite profiling in metabonomics/metabolomics. TrAC Trend. Anal. Chem. 27, 251–260 (2008).

[b20] Naz S., Vallejo M., García A. & Barbas C. Method validation strategies involved in non-targeted metabolomics. J. Chromatogr. A 1353, 99–105 (2014). 24811151 10.1016/j.chroma.2014.04.071

[b21] Xia J., Sinelnikov I. V., Han B. & Wishart D. S. MetaboAnalyst 3.0-making metabolomics more meaningful. Nucleic Acids Res. 43, W251–257 (2015). 25897128 10.1093/nar/gkv380 PMC4489235

[b22] Zhang W., Li F. & Nie L. Integrating multiple ‘omics’ analysis for microbial biology: application and methodologies. Microbiology 156, 287–301 (2010). 19910409 10.1099/mic.0.034793-0

[b23] Henderson J. F. & Paterson A. R. P. Nucleotide metabolism: an introduction. (Academic Press, 2014).

[b24] Mathis D. & Shoelson S. E. Immunometabolism: an emerging frontier. Nat. Rev. Immunol. 11, 81–83 (2011). 21469396 10.1038/nri2922 PMC4784680

[b25] Silva N. M. *et al.* Expression of indoleamine 2, 3-dioxygenase, tryptophan degradation, and kynurenine formation during *in vivo* infection with *Toxoplasma gondii*: induction by endogenous gamma interferon and requirement of interferon regulatory factor 1. Infect. Immun. 70, 859–868 (2002). 11796621 10.1128/iai.70.2.859-868.2002 PMC127654

[b26] Leonard B. & Maes M. Mechanistic explanations how cell-mediated immune activation, inflammation and oxidative and nitrosative stress pathways and their sequels and concomitants play a role in the pathophysiology of unipolar depression. Neurosci. Biobehav. Rev. 36, 764–785 (2012). 22197082 10.1016/j.neubiorev.2011.12.005

[b27] Dantzer R., O’Connor J. C., Lawson M. A. & Kelley K. W. Inflammation-associated depression: from serotonin to kynurenine. Psychoneuroendocrinology 36, 426–436 (2011). 21041030 10.1016/j.psyneuen.2010.09.012 PMC3053088

[b28] Wonodi I. & Schwarcz R. Cortical kynurenine pathway metabolism: a novel target for cognitive enhancement in schizophrenia. Schizophr. Bull. 36, 211–218 (2010). 20147364 10.1093/schbul/sbq002 PMC2833131

[b29] Cai X. *et al.* Local potentiation of excitatory synapses by serotonin and its alteration in rodent models of depression. Nat. Neurosci. 16, 464–472 (2013). 23502536 10.1038/nn.3355 PMC3609911

[b30] Prandovszky E. *et al.* The neurotropic parasite *Toxoplasma gondii* increases dopamine metabolism. PLoS One 6, e23866 (2011). 21957440 10.1371/journal.pone.0023866 PMC3177840

[b31] Stibbs H. Changes in brain concentrations of catecholamines and indoleamines in *Toxoplasma gondii* infected mice. Ann. Trop. Med. Parasitol. 79, 153–157 (1985). 2420295 10.1080/00034983.1985.11811902

[b32] Ferguson D. & Hutchison W. An ultrastructural study of the early development and tissue cyst formation of *Toxoplasma gondii* in the brains of mice. Parasitol. Res. 73, 483–491 (1987). 3422976 10.1007/BF00535321

[b33] Xiao J. F. *et al.* LC–MS based serum metabolomics for identification of hepatocellular carcinoma biomarkers in Egyptian cohort. J. Proteome Res. 11, 5914–5923 (2012). 23078175 10.1021/pr300673x PMC3719870

[b34] Peng J., Chen Y. T., Chen C. L. & Li L. Development of a universal metabolome-standard method for long-term LC–MS metabolome profiling and its application for bladder cancer urine-metabolite-biomarker discovery. Anal. Chem. 86, 6540–6547 (2014). 24877652 10.1021/ac5011684

[b35] Huybrechts B., Martins J., Debongnie P., Uhlig S. & Callebaut A. Fast and sensitive LC-MS/MS method measuring human mycotoxin exposure using biomarkers in urine. Arch. Toxicol. 89, 1993–2005 (2014). 25209565 10.1007/s00204-014-1358-8

[b36] Xu D. D. *et al.* Discovery and identification of serum potential biomarkers for pulmonary tuberculosis using iTRAQ-coupled two-dimensional LC-MS/MS. Proteomics 14, 322–331 (2014). 24339194 10.1002/pmic.201300383

[b37] Li J. V. *et al.* Metabonomic investigation of single and multiple strain *Trypanosoma brucei brucei* infections. Am. J. Trop. Med. Hyg. 84, 91–98 (2011). 21212208 10.4269/ajtmh.2011.10-0402 PMC3005522

[b38] Balog C. I. *et al.* Metabonomic investigation of human *Schistosoma mansoni* infection. Mol. Biosyst. 7, 1473–1480 (2011). 21336380 10.1039/c0mb00262c

[b39] Lakshmanan V. *et al.* Metabolomic analysis of patient plasma yields evidence of plant-like α-linolenic acid metabolism in *Plasmodium falciparum*. J. Infect. Dis. 206, 238–248 (2012). 22566569 10.1093/infdis/jis339 PMC3490690

[b40] Hill D. E., Chirukandoth S., Dubey J., Lunney J. K. & Gamble H. Comparison of detection methods for *Toxoplasma gondii* in naturally and experimentally infected swine. Vet. Parasitol. 141, 9–17 (2006). 16815636 10.1016/j.vetpar.2006.05.008

[b41] Wikoff W. R. *et al.* Metabolomics analysis reveals large effects of gut microflora on mammalian blood metabolites. Proc. Natl. Acad. Sci. USA. 106, 3698–3703 (2009). 19234110 10.1073/pnas.0812874106 PMC2656143

[b42] Zelena E. *et al.* Development of a robust and repeatable UPLC-MS method for the long-term metabolomic study of human serum. Anal. Chem. 81, 1357–1364 (2009). 19170513 10.1021/ac8019366

[b43] Luan H. *et al.* Pregnancy-induced metabolic phenotype variations in maternal plasma. J. Proteome Res. 13, 1527–1536 (2014). 24450375 10.1021/pr401068k

[b44] Smith C. A., Want E. J., O’Maille G., Abagyan R. & Siuzdak G. XCMS: processing mass spectrometry data for metabolite profiling using nonlinear peak alignment, matching, and identification. Anal. Chem. 78, 779–787 (2006). 16448051 10.1021/ac051437y

[b45] Guo L. *et al.* Three plasma metabolite signatures for diagnosing high altitude pulmonary edema. Sci. Rep. 5, 15126; 10.1038/srep15126 (2015). 26459926 PMC4602305

[b46] Wiklund S. *et al.* Visualization of GC/TOF-MS-based metabolomics data for identification of biochemically interesting compounds using OPLS class models. Anal. Chem. 80, 115–122 (2008). 18027910 10.1021/ac0713510

[b47] Xu Y. *et al.* Evaluation of accurate mass and relative isotopic abundance measurements in the LTQ-orbitrap mass spectrometer for further metabolomics database building. Anal. Chem. 82, 5490–5501 (2010). 20515063 10.1021/ac100271j

