# Supplementary material for: Metabolomic Profiling of Mice Serum during Toxoplasmosis Progression Using Liquid Chromatography-Mass Spectrometry
Source: Sci Rep. 2016 Jan 20;6:19557. doi: 10.1038/srep19557 (PMC4726199; doi:10.1038/srep19557)

**Supplementary information**

**Metabolomic Profiling of Mice Serum during Toxoplasmosis Progression Using Liquid Chromatography-Mass Spectrometry**

Chun-Xue Zhou^1, 2¶^, Dong-Hui Zhou^1¶^, Hany M. Elsheikha^3^, Yu Zhao^1^, Xun Suo^2*^&

Xing-Quan Zhu^1*^

^1^State Key Laboratory of Veterinary Etiological Biology, Key Laboratory of Veterinary Parasitology of Gansu Province, Lanzhou Veterinary Research Institute, Chinese Academy of Agricultural Sciences, Lanzhou, Gansu Province 730046, PR China, ^2^National Animal Protozoa Laboratory and College of Veterinary Medicine, China Agricultural University, Beijing 100193, PR China, ^3^Faculty of Medicine and Health Sciences, School of Veterinary Medicine and Science, University of Nottingham, Sutton Bonington Campus, Loughborough, LE12 5RD, UK

__________________________________________________________________

^¶^These authors contributed equally to this work.

Correspondence and requests for materials should be addressed to X.Q.Z. (email: xingquanzhu1@hotmail.com) or X.S. (email: suoxun@cau.edu.cn)

**Figure S1.** **The size of spleen of *T. gondii*-infected mice (left) compared to that of mock-infected controls (right).**


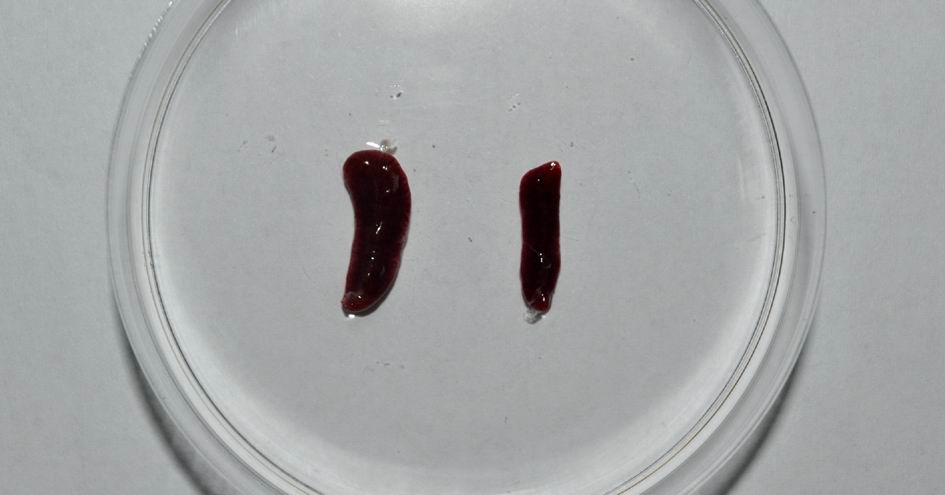


**Figure S2. Overlay of all total ion current (TIC) chromatograms for serum samples obtained in the (A) positive ion mode (ESI+) and (B) negative ion mode (ESI-).** Y-axis represents the intensity.

**
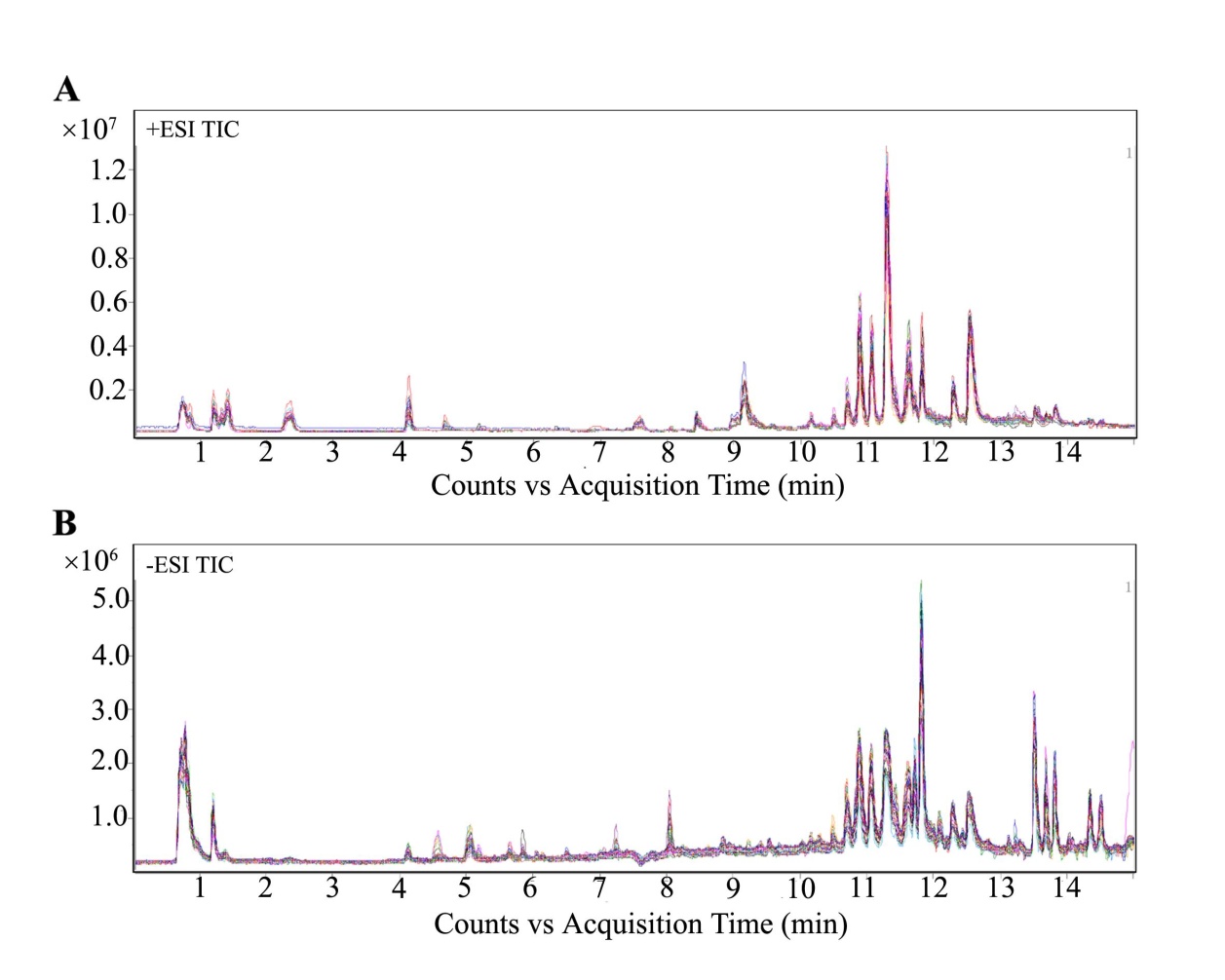
**

**Figure S3.** **Principle component analysis (PCA) scores plot of *T. gondii*-infected mice, normal mice and quality control samples based on serum LC-Q-TOF-MS data.** PCA scores plots for metabolite profiles obtained in (A) positive ion mode (ESI+) and (B) negative ion mode (ESI-).

**
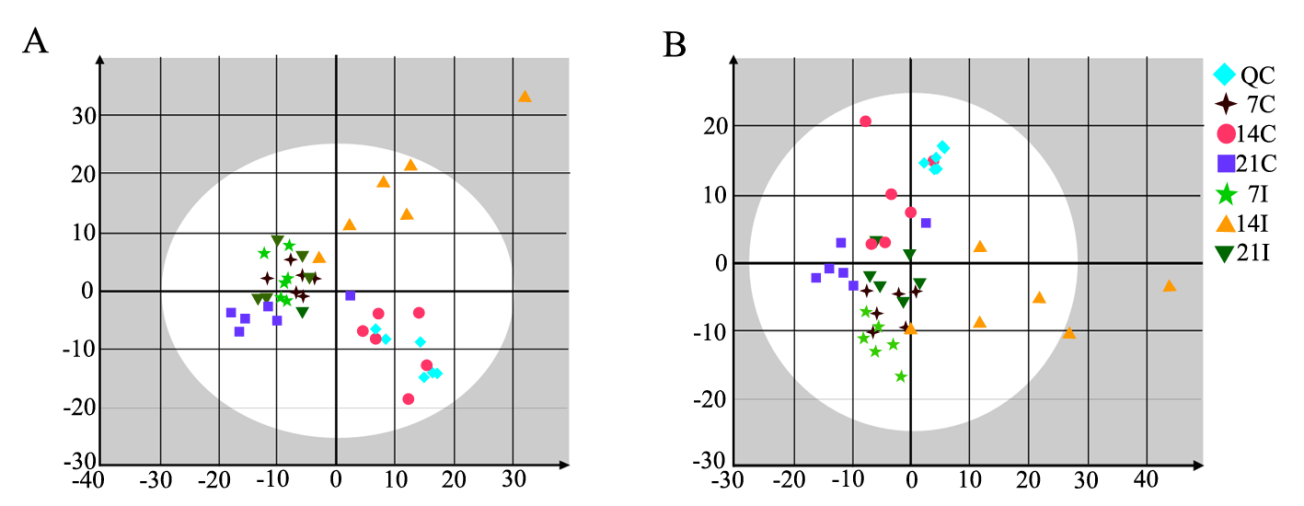
**

**Figure S4.** **Differential metabolic profile in mice serum among the six mice groups and during the time courses of *T. gondii* infection.** (A) 2D PCA score plot (ESI+) depicting time-dependent trajectory of metabolic profile at three time points (7, 14 and 21 day) after *T. gondii* infection; (B) 2D PCA score plot (ESI-).


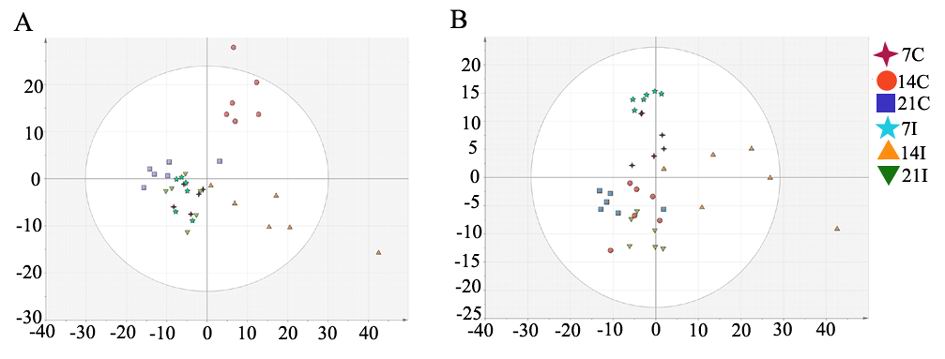


**Figure S5**. **Heat map representation of the significantly changed metabolites found between infected groups and the corresponding controls in the ESI- mode.** (A) 7Dinfected group vs 7D control; (B) 14D infected group vs 14D control; (C) 21D infected group vs 21D control.

**
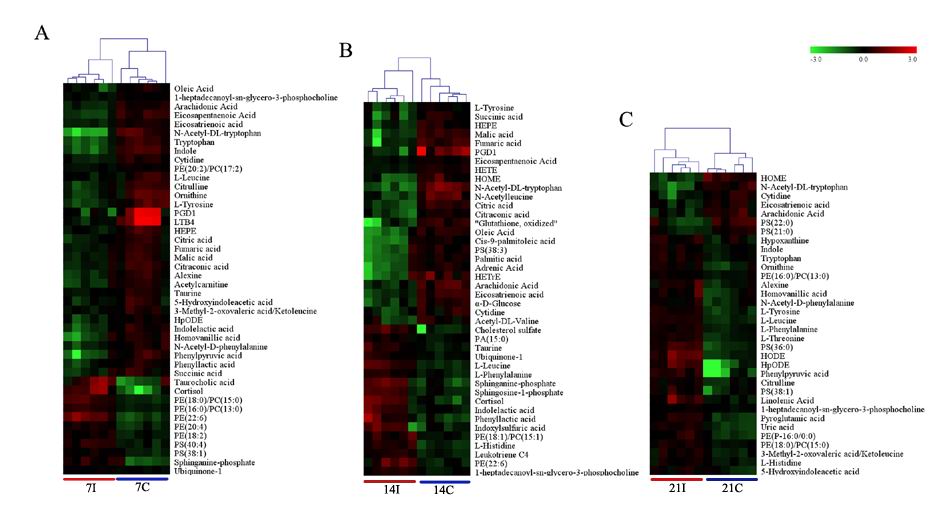
**

**Figure S6.** **Heat map representation of the significantly changed metabolites found between different infected groups in the ESI- mode.** (A) 7D infected group vs 21D infected group; (B) 7D infected group vs 14D infected group; (C) 14D infected group vs 21D infected group.

**
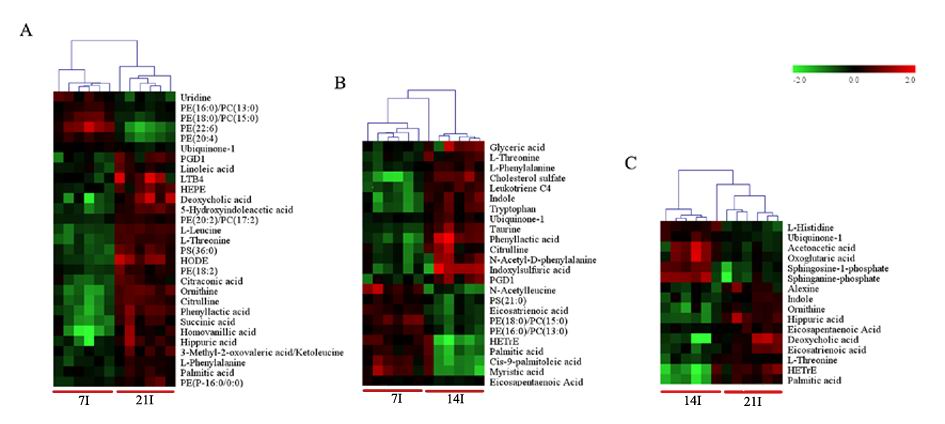
**

**Figure S7**. **Unsupervised hierarchical clustering plot for infected and control mice groups in (A) ESI+ mode, (B) ESI- mode.** Individual samples (horizontal axis) and compounds (vertical axis) are separated using hierarchical clustering. Red color indicates control samples, green color indicates infected samples. As shown in the heat map alignment, mice groups were clustered by an unsupervised algorithm, which confirms the presence of discriminating features between the infected and control groups.


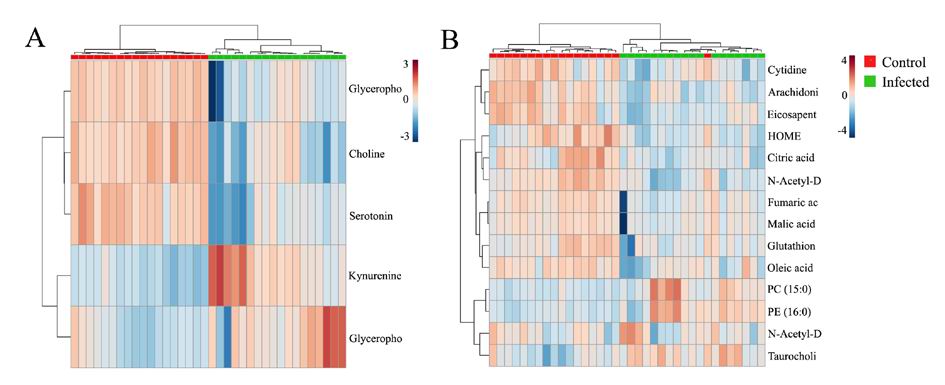

Supplement: Supplementary Information [file srep19557-s1.docx]
